# Quercetin-mediated restoration of high-fat diet-induced male reproductive dysfunction through modifying spermatogenesis and unraveling 3β-HSD, 17β-HSD, and StAR pathways

**DOI:** 10.1186/s40360-025-00918-y

**Published:** 2025-04-22

**Authors:** Mona H. Hafez, Shereen B. Gad, Yasser S. El-Sayed

**Affiliations:** 1 Physiology Department, Faculty of Veterinary Medicine, Alexandria University, Alexandria, 22758 Egypt; 2 Veterinary Forensic Medicine and Toxicology Department, Faculty of Veterinary Medicine, Damanhour University, Damanhour, Egypt

**Keywords:** Quercetin, High-fat diet, Testicular function, Steroidogenic enzymes, Antioxidant system, Androgen synthesis

## Abstract

**Purpose:**

We explored the astounding potential of quercetin (QRT) to counteract the determinantal impacts of a high-fat diet (HFD) on testicular function in rat model. The goal was to understand how QRT, and its mechanisms of action can protect testicular health from HFD.

**Methods:**

Rats were divided into four groups receiving a control diet, QRT supplement (100 mg/kg), HFD, or HFD plus QRT for 8 weeks. Afterward, assessments were conducted, and reproductive organs were analyzed for hormone levels, gene expression, and subjected to biochemical, histopathological, and immunohistochemical analyses.

**Results:**

The HFD caused substantial declines in testicular weight, accessory sex glands and epididymis. The HFD also negatively impacted sperm characteristics including reduced motility, viability, and count, along with impaired morphology. Additionally, the HFD decreased testosterone levels in the testes and serum, impaired antioxidant enzymes like superoxide dismutase (SOD), glutathione peroxidase (GPx), and catalase, and reduced expression of key steroid metabolism enzymes 17β-hydroxysteroid dehydrogenase (17β-HSD), 3β-hydroxysteroid dehydrogenase (3β-HSD), and steroidogenic acute regulatory protein (StAR) involved in testosterone synthesis. These changes were paired with enhanced testicular lipid peroxidation, nitrite, and the inflammatory marker tumor necrosis factor-alpha (TNF-α), reflecting the damaging еffеcts of the HFD. Examination of testicular tissues verified structural damage and significantly fewer proliferating cell nuclear antigen (PCNA)-positive spermatogenic cells in seminiferous tubules of HFD-fed group, confirming HFD’s adverse еffеcts.

**Conclusion:**

QRT supplementation was able to curb the harmful impacts of the HFD on testicular spermatogenesis and steroidogenesis through its antioxidant, anti-inflammatory and androgen boosting properties.

## Introduction

In recent years, research has unveiled a striking connection between lipid metabolism and male reproductive health. Epidemiological data has consistently highlighted the repercussions of high-fat diets (HFDs), showing a significant enhancement in the rate of metabolic diseases, counting cardiovascular issues, obesity, and non-alcoholic fatty liver disease [1, 2]. Surprisingly, these diets have been also linked to reproductive dysfunction, raising questions about the intricate relationship between nutrition and fertility. The ubiquity of HFDs is not confined to specific income brackets or regions; it is a global phenomenon [3]. As these diets become increasingly common, researchers are taking note of their profound impact on our reproductive health. Recent research has provided compiling evidence connecting HFDs and irregular lipid metabolism with significant variations in fertility and semen quality [4]. To understand this link, we must first delve into the role of cholesterol. It, often associated with heart health, serves as an indigenous component crucial for the structure and function of cell membranes. It goes beyond this role, however, as it stands as the precursor for the biosynthesis of steroid hormones. This dual importance underscores the significance of maintaining cholesterol homeostasis, particularly concerning male reproductive function. Any disruption in this balance can potentially exert negative еffеcts on male reproduction [5].

The consequences of HFDs on male reproductive function are far-reaching. These diets trigger a cascade of issues, including but not limited to low serum testosterone levels, metabolic chaos in sexual hormones; reproductive cell apoptosis and poor sperm quality, which can have profound implications for fertility [5, 6]. As testosterone levels decrease due to dietary factors, they exacerbate the metabolic syndrome, setting in motion a vicious cycle [7]. Testosterone, the chief androgen produced in the Leydig cells, partake a fundamental role in spermatogenesis and the development of male reproductive organs. Various key proteins participate in the formation of testosterone, including steroidogenic acute regulatory protein (StAR) and cholesterol side-chain cleavage enzyme (P450scc), which breakdown pregnenolone, a precursor to testosterone [8, 9]. Once pregnenolone is formed, it is transferred to smooth endoplasmic reticulum by 3β-hydroxysteroid dehydrogenase (3β-HSD) and subsequently reduced by 17β-hydroxysteroid dehydrogenase (17β-HSD) to synthesis testosterone [10]. These intricate processes underscore how sensitive the system is to disruptions, such as those caused by HFDs. Studies have presented compelling evidence that HFD intake and obesity conspire to down-regulate the expression of StAR and P450scc, culminating in a distressing impact on reproductive function [11]. Despite these crucial insights, the precise molecular mechanisms explaining harmful impacts of dietary fat overloads on the fertility of male remain elusive [4].

High-fat diets have been found to induce systemic oxidative stress, unleashing a wave of consequences that jeopardize quality of male gametes and consequently, fertility. Sperm cells, which are rich in polyunsaturated fatty acids, have a pivotal role in reproductive sequences and normal physiology of sperms. However, they also become a goal for oxidative damage in the presence of oxidative stress [12], that еmеrgеs as a central player in the narrative of male reproductive health. The mechanism of programmed deterioration has far-reaching consequences, extending to deprived fertilization, compromised embryonic development, abortion, birth defects (including autism), and even childhood cancer [13]. Deleterious impact of oxidative stress underscores the urgent need to explore interventions that can mitigate its еffеcts.

Amidst this challenging scenario, a naturally occurring food flavonoid called quercetin (QRT; pentahydroxy flavone) еmеrgеs as a potential savior. It is found in various edible plants, including vegetables, fruits, and grains. Its biological potential is attributed to its composition of five hydroxyl groups [14]. This flavonoid boasts an impressive array of characteristics, counting anti-inflammatory, antioxidant, antimicrobial, and anti-allergic activities. It also offers hepatoprotective and neuroprotective еffеcts, thanks to its potent antioxidant and frее-radical scavenging capabilities [15]. Quercetin’s role in preserving male reproductive health is further underscored by its ability to improve sperm quality. Studies have shown that quercetin supplementation can enhance sperm quality, offering a promising advantage for increasing men’s fertility rates [14]. This еffеcts is mostly important given the vulnerability of polyunsaturated fatty acids-rich sperm to oxidative damage. Quercetin’s protective abilities extend beyond its antioxidant properties. It acts as an enzyme-mimetic antioxidant, reducing pro-inflammatory cytokine expression and protecting cells against excessive apoptosis [15, 16]. These additional mechanisms make QRT a formidable defender against the harmful impacts of HFD-induced oxidative damage on male reproductive functions. Given these promising attributes, the aim of the recent experiment was to examine the impact of HFD on male reproductive functions and explore potential ameliorative role of quercetin in preserving male steroidogenic and reproductive functions under such challenging dietary conditions by measuring the relative weights of reproductive organs, testosterone hormone levels, sperm characteristics, steriodogenic enzymes gene expression (3β-HSD, 17β-HSD, and StAR), oxidative/antioxidant status, histological alterations, and immunohistochemical staining of PCNA in rat’s testicular tissue.

## Materials and methods

### Quercetin source

Quercetin hydrate with a minimum purity of 97%, in the form of a powder, was acquired from Sigma-Aldrich, USA [Product #: 337951, Pack size: 100 g].

### Animal subjects, experimental setup and group allocation

In this study, 32 male Sprague–Dawley (SD) rats, with a weight range of 150–200 g, were procured from Medical Research Institute’s animal breeding unit at Alexandria University, Egypt. The rats had an average age of 8–9 months at the onset of the study. To ensure the consistency of the experimental conditions, the animals were subjected to constant nutritional and environmental settings. They were kept in a controlled environment at room temperature, with a 12-hour light-on and 12-hour light-off schedule.

To ensure a comprehensive and methodical study, the rats were thoughtfully distributed into individual, well-ventilated propylene cages. Each of these cages was equipped with metal mesh covers, supplying ample aeration to the rats. Each cage housed a group of five rats, facilitating their observation and care. The animals were kept for 2 weeks of acclimatization. The rats were systematically organized and assigned into four distinct groups, each comprising eight rats. This division was maintained over a total duration of 8 weeks, following the protocol outlined in the study by Rasheed, Elshikh [12]:

- **Group I (Control)**: The group was considered as the control group and was provided with a standard ration, which was formed from 13.5% fat, 61.3% carbohydrates, and 25.2% protein, with a total energy value of 2830 kcal/kg [2]. Each rat received 0.5ml 0.1% Tween-80 in distilled water (vehicle of QRT) by oral gavage.
- **Group II (QRT)**: Rats in this group were given the same standard diet as the control group, but with the addition of QRT at a dosage of 100 mg/kg bwt daily dissolved in 0.1% Tween-80 in distilled water by oral gavage [14].
- **Group III (HFD)**: This group was given an HFD, comprising of 60% fat, 20% carbohydrates, 20% protein and 5243 kcal/kg energy content. Notably, the HFD also included 232 mg of cholesterol/g [17]. Each rat received 0.5 ml 0.1% Tween-80 in distilled water (vehicle of QRT) by oral gavage.
- **Group IV (QRT + HFD)**: Rats in this group were subjected to the same HFD as Group III. However, in addition to the HFD, they were administered QRT at a dosage of 100 mg/kg bwt daily dissolved in 0.1% Tween-80 in distilled water by oral gavage.

### Samples assembly and preparation

After ending the 8-wееk experimental period, a comprehensive assessment was conducted on all participating rats. Their weights were recorded, marking the end of this stage of the study. Subsequently, the rats were humanely euthanized through the administration of anesthesia overdose, specifically 100 mg/kg of ketamine-xylazine via intraperitoneal injection [9]. Serum samples were meticulously obtained by subjecting collected blood samples to centrifugation at 1,957 × g for 10 min for later analysis, primarily for the quantification of reproductive hormone levels, specifically testosterone.

In a meticulous procedure, samples of testes, epididymis, and accessory sex glands were extracted and weighed. Left testis, a crucial focus of the study, underwent further division into two distinct segments. The first segment was subjected to homogenization, resulting in the creation of a 10% homogenate (weight/volume) using ice-cold 0.1 M Tris (hydroxymethyl) aminomethane-HCl (Tris-HCl), maintaining a pH of 7.4. This homogenization process was meticulously executed within an ice-chilled glass-homogenizing vessel, using a homogenizer fitted with a Teflon pestle from Glass-Col in the United States.

After homogenization, a cooling centrifuge was employed to subject the homogenate to centrifugation at 3000 ×g for 10 min at 4 ºC. The objective was to effectively eliminate nuclei and debris thereby isolating the supernatant. This supernatant, a crucial specimen, was then stored at a temperature of -80 ºC. This stored supernatant proved to be an invaluable resource for a range of assessments, including the evaluation of intra-testicular testosterone levels, testicular antioxidant enzymatic system (encompassing superoxide dismutase, catalase, and glutathione peroxidase), levels of oxidative/nitrosative stress markers (including the lipid peroxidation indicator malondialdehyde and nitrite), and levels of the tumor necrosis factor-alpha (TNF-α, pro-inflammatory cytokine). The second segment of the left testicular tissue served as the foundation for the examination of gene expression related to steroidogenesis (3β-HSD, 17β-HSD, and StAR) which was placed in liquid nitrogen, then stored at − 80 ◦C until analysis.

Right testes, on the other hand, were reserved for histopathological and immunohistochemical analyses. To facilitate these assessments, they were preserved in a solution of 10% neutral-buffered paraformaldehyde solution.

### Reproductive organ index weight (IW)

Using a Mettler balance P1210 (Mettler Instruments AG, Switzerland) with a sensitivity of 0.01 g, the reproductive tissues were weighed to assess the relative weight, which include the testes, accessory sex glands (seminal vesicles and prostate), and epididymis, an index known as the Index Weight (IW) was calculated [18]. The formula for calculating the index weight (IW) is as follows: IW = 100 × [organ weight (g) / body weight (g)]

### Evaluation of epididymal spermatozoa

The evaluation of epididymal spermatozoa involved a comprehensive analysis of three key parameters: count, motility, and abnormalities. In addition to the determination of the epididymal sperms concentration and spermatozoal viability. The methodology employed for these assessments were adapted from the approach outlined by Sonmez, Turk [19]. A total of 200 spermatozoa from each rat were examined and individually scored normal or abnormal, according to the strict sperm morphology criteria. The morphological abnormalities were divided into head and tail defects. The percentages of abnormal shaped sperms were calculated.

### Testosterone assessment with ELISA

The quantification of serum and intra-testicular testosterone values was performed by the Enzyme-Linked Immunosorbent Assay (ELISA) technique, consuming commercial ELISA kits (Immunotеch Beckman Coulter Co., USA) following the provided manufacturer’s instructions.

### Estimation of testicular antioxidant enzymes and oxidative/nitrosative stress markers

The determination of testicular antioxidant enzymatic system activities, including superoxide dismutase (SOD) [20], catalase [21], and glutathione peroxidase (GPx) [22] was executed through the employment of a spectrophotometric approach. Furthermore, the evaluation of oxidative/nitrosative stress markers within testicular tissue homogenates involved the assessment of the lipid peroxidation indicator malondialdehyde (MDA) [23] and the presence of nitrite, through the utilization of analytical assay kits (Bio diagnostic Co., Cairo, Egypt). Nitric oxide levels, on the other hand, were indirectly determined through the measurement of nitrite production using the Griess diazotization reaction [24]. Lastly, the protein content within the testicular tissues was quantified [25].

### Measurement of pro-inflammatory cytokine (TNF-α)

Assessment of the pro-inflammatory cytokine TNF-α was conducted within the homogenized testicular tissue samples. This measurement was carried out in strict adherence to the protocols specified by the manufacturer of the purchased ELISA kits [26]. An ELISA Plate Reader from Bio-Rad in Hercules, CA, USA, was employed for the final readings.

### Testicular steroidogenic enzymes gene expression

#### RNA extraction and reverse transcription

A total of 30 mg of testicular tissue of seven rats per group was employed for the extraction of total RNA. The extraction process adhered to the protocol detailed in the RNеasy Mini Kit from Qiagen in Heidelberg, Germany. The purity of the extracted total RNA was assessed using the Nanodrop^®^ ND-1000 Spectrophotometer from Nano-Drop Technologies in Wilmington, DE, USA. This assessment ensured the quality and integrity of the RNA samples. To facilitate the analysis of gene expression, 0.5 µg of the total RNA was subjected to Reverse transcription, leading to the synthesis of complementary DNA (cDNA). This process was accomplished using the QIAGEN Long Range 2 Step RT-PCR Kit, following the manufacturer’s instructions.

For subsequent analysis, one µl of the resulting cDNA was mixed with 12.5 µl of 2x SYBR^®^ Green PCR mix with ROX from Bio-Rad, along with 5.5 µl of autoclaved water. Additionally, 0.5 µl of each forward and Reverse primers for the target genes were included in the mix. Expression levels were normalized using an internal housekeeping control, the β-actin gene.

#### Real-time PCR

The expressions of Specific steroidogenic genes, including 3β-hydroxysteroid dehydrogenase type 6 (3β-HSD6), 17β-hydroxysteroid dehydrogenase type 3 (17β-HSD3), and StAR, were evaluated using Real-time PCR. Specific primer sequences for the target genes were utilized in the analysis (see Table 1).

**Table 1:** Oligonucleotide primer sequences of steroidogenic genes

Gene	Accession number	Primer sequence (5′- 3′)		Amplicon size (bp)
		Forward primer	Reverse primer	
3β-HSD6	M38178	TGTGCCAGCCTTCATCTAC	CTTCTCGGCCATCCTTTT	145
17β-HSD3	NM_054007	GACCGCCGATGAGTTTGT	TTTGGGTGGTGCTGCTGT	140
StAR	NM_0315583	GGGCATACTCAACAACCAG	ACCTCCAGTCGGAACACC	111
β-actin	NM_007393	TCACTATCGGCAATGTGCGG	GCTCAGGAGGAGCAATGATG	156

These primers are used for PCR amplification of specific gene regions, and the resulting amplicon sizes are indicated. The genes are 3β-hydroxysteroid dehydrogenase type 6 (3β-HSD6), 17β-hydroxysteroid dehydrogenase type 3 (17β-HSD3), steroidogenic acute regulatory protein (StAR), and β-actin

The PCR reactions were performed using a Rotor-Gene Q cycler from Qiagen. The program consisted of an initial enzyme activation step at 94 °C for 2 min, followed by 40 cycles of denaturation at 95 °C for 15 s, annealing at 60 °C for 30 s, and extension at 72 °C for 30 s. Fluorescent product detection occurred at end of the extension phase. Amplification data were gathered by sequence detector and analyzed using sequence detection software. For every analyze, a standard curve was made using varying amounts of cDNA. The slope of these curves verified appropriate PCR circumstances (slopes typically falling within the range of 3.3–3.6). The concentration of RNA in every sample was assessed based on threshold cycle (Ct) values, examined using software supplied by the manufacturer. Quantification of fold changes in mRNA expression was performed in relation to β-actin mRNA values in the corresponding experimental groups. This calculation was achieved using the 2^−ΔΔ^Ct method.

### Histopathological examination

The right testis of seven rats per group was subjected to fixation using a 10% paraformaldehyde solution. Following fixation, the testis was embedded in paraffin, resulting in the creation of testes blocks. Serial sections of the blocks, each measuring 5 μm in thickness, were meticulously cut utilizing a rotary microtome. These sections were intended for subsequent hematoxylin and eosin (H&E) staining and histopathological examination. Paraffin sections were initially dewaxed. Subsequently, a brief 3-second hematoxylin staining was conducted, followed by a 3-min eosin staining. The sections then underwent a process of dehydration using alcohol and clearing with xylene. The sections were sealed. The stained sections were subjected to scrutiny under a light microscope. Detailed observations of histopathological lesions were carried out, and relevant findings were documented. This examination was supported by the use of a digital camera (Nikon Corporation Co., Ltd., Japan) for photographic documentation [27].

### Immunohistochemical evaluation of PCNA antibodies

Testicular sections were dewaxed and then treated with a solution of 0.05 M citrate buffer (pH 6.8) for antigen retrieval. Following antigen retrieval, the sections were subjected to a treatment involving 0.3% H_2_O_2_ and protein block. These steps were vital for optimal immunohistochemical staining. The sections were subsequently incubated with an antibody targeting proliferating cell nuclear antigen (PCNA). This Specific antibody, designated as monoclonal mousе-PC10 (MAS-11358, Invitrogen), was used at a dilution of 1:200. After rinsing with PBS, the sections were exposed to an anti-mouse secondary antibody, Specifically the EnVision+™ System Horsеradish Peroxidase Labeled Polymer by Dako. The incubation was conducted for 30 min at room temperature. Visualization of the immunohistochemical reaction was achieved using a DAB kit. Finally, Mayer’s hematoxylin was employed as a counterstain to enhance the contrast of the stained sections. The expression of PCNA was quantified as a pеrcеntagе of positive expression among a total of 1000 cells within 8 high-power fields (HPF) [28].

### Statistical analysis

Obtained data were revealed as the mean ± standard deviation (SD). To analyze the statistical significance of results across various experimental groups, a one-way analysis of variance (ANOVA) was conducted. Subsequently, post hoc analysis was carried out using Duncan’s test to facilitate comparisons between different experimental groups. These statistical procedures were executed employing IBM SPSS Statistics computer software (version 21). Statistical significance was determined by a threshold of P-values < 0.05, indicating results that hold considerable statistical importance.

## Results

### Reproductive organ index weights

Impact of HFD on reproductive organ index weights was a vital focus of this study. Notably, the consumption of HFD led to a marked (*P* < 0.05) decrease in relative weights of reproductive organs relative to the control and QRT-treated rats. Intriguingly, QRT administration improved all these criteria, effectively restoring them to normal levels (Table 2).

**Table 2:** Effect of quercetin (QRT) on the relative index weights (IW) of different reproductive organs in high-fat diet (HFD)-induced male rats

Treatment	Testes IW	Epididymis IW	Accessory sex glands IW
Control	1.205 ± 0.10 ^b^	0.549 ± 0.033 ^a^	0.992 ± 0.058 ^a^
QRT	1.309 ± 0.09 ^a^	0.579 ± 0.024 ^a^	0.983 ± 0.045 ^a^
HFD	0.417 ± 0.03 ^d^	0.125 ± 0.009 ^c^	0.302 ± 0.028 ^c^
QRT + HFD	0.814 ± 0.042 ^c^	0.409 ± 0.021 ^b^	0.708 ± 0.059 ^b^

Note: Values are expressed as mean ± standard deviation (SD) (*n* = 8). Means within the same column without a common superscript letter differ significantly (*P* < 0.05)

### Sperm characteristics

The study also delved into sperm characteristics, providing critical insights into the impact of HFD on reproductive function. HFD induced a state of reproductive dysfunction, as evidenced by a marked reduction (*P* < 0.05) in various sperm characteristics. This includes epididymal sperm count, both mass and individual motility, the pеrcеntagе of viable sperm, as well as a substantial enhancement in sperm anomalies relative to control group (Table 3).

**Table 3:** Effect of quercetin (QRT) on sperm characteristics of high-fat diet (HFD)-fed male rats

Treatment	Sperm count (million/ml)	Mass sperm motility (%)	Individual sperm motility (%)	Sperm abnormalities	Alive sperm (%)
Control	254.00 ± 18.31 ^a^	89.25 ± 5.33 ^a^	71.11 ± 4.82 ^a^	8.14 ± 0.87 ^c^	88.21 ± 4.35 ^a^
QRT	267.63 ± 14.29 ^a^	91.16 ± 6.08 ^a^	73.83 ± 5.82 ^a^	7.91 ± 0.91 ^c^	92.45 ± 6.74 ^a^
HFD	122.37 ± 8.38 ^c^	42.64 ± 2.72 ^c^	32.55 ± 1.83 ^c^	27.Tret 1.83 ^a^	52.82 ± 3.95 ^c^
QRT + HFD	193.31 ± 14.62 ^b^	72.33 ± 4.02 ^b^	58.21 ± 3.75 ^b^	12.84 ± 1.06 ^b^	75.62 ± 4.71 ^b^

Note: Values are expressed as mean ± standard deviation (SD) (*n* = 8). Means within the same column without a common superscript letter differ significantly (*P* < 0.05)

QRT intervention yielded noteworthy improvements, which led to a marked improvement in sperm count relative to HFD group. Additionally, it enhanced both mass and individual sperm motility and enhanced the pеrcеntagе of viable sperm while reducing sperm abnormalities. Notably, these improvements brought the sperm characteristics close to levels observed in the control group.

### Serum and intra-testicular testosterone

Investigations into serum and intra-testicular testosterone hormone values is presented in Table 4, highlighting crucial observations among the different experimental groups. Data revealed a marked (*P* < 0.05) lowing in both serum and intra-testicular testosterone hormone levels in HFD-fed rats compared to control and QRT-treated groups. The administration of QRT to HFD-fed rats led to a substantial improvement in testosterone levels, relative to the rats subjected solely to HFD. This marked enhancement in reproductive hormone levels suggested that QRT affectively mitigated the reproductive dysfunction observed in HFD rats.

**Table 4:** Effect of quercetin (QRT) on serum and intra-testicular testosterone levels in high-fat diet (HFD)-fed male rats

Treatment	Serum Testosterone (ng/ml)	Intra-testicular testosterone (ng/mg protein)
Control	3.32 ± 0.38 ^b^	15.45 ± 1.13 ^b^
QRT	3.68 ± 0.43 ^a^	21.29 ± 1.82 ^a^
HFD	0.85 ± 0.05 ^d^	6.21 ± 0.28 ^d^
QRT + HFD	2.04 ± 0. 18 ^c^	11.32 ± 1.07 ^c^

Note: Values are expressed as mean ± standard deviation (SD) (*n* = 8). Means within the same column without a common superscript letter differ significantly (*P* < 0.05)

### Testicular oxidative/nitrosative status

The evaluation of testicular oxidative/nitrosative status is detailed in the context of Table 5, revealing significant insights into the impact of HFD and QRT treatment. The results demonstrated that HFD-fed rats displayed a marked (*P* < 0.05) enhancement in testicular MDA and nitrite levels. This increase was accompanied by a notable lowering in activities of SOD, catalase, and GPx, relative to normal control rats. QRT alleviation resulted in a reduction in the elevated lipid peroxidation and nitrosative biomarkers caused by HFD. Moreover, activities of antioxidant enzymes demonstrated a partial restoration in HFD + QRT treated group in comparison with the control.

**Table 5:** Effect of quercetin (QRT) on testicular oxidative/nitrosative parameters and proinflammatory cytokine levels in high-fat diet (HFD)-fed male rats

Treatment	MDA (nmol/mg protein)	Nitrite (µmol/mg protein)	SOD (U/mg protein)	GPx (U/mg protein)	CAT (U/mg protein)	TNF-α (pg/mg protein)
Control	1.89 ± 0.12 ^c^	1.69 ± 0.07 ^c^	6.02 ± 0.21 ^b^	7.03 ± 0.28 ^b^	7.96 ± 0.32 ^a^	6.92 ± 0.31 ^c^
QRT	1.29 ± 0.07 ^d^	1.41 ± 0.03 ^d^	7.24 ± 0.32 ^a^	10.68 ± 1.01 ^a^	8.08 ± 0.45 ^a^	5.24 ± 0.27 ^d^
HFD	8.31 ± 0.48 ^a^	9.14 ± 0.53 ^a^	1.67 ± 0.08 ^d^	2.12 ± 0.12 ^d^	2.05 ± 0.06 ^c^	15.38 ± 1.28 ^a^
QRT + HFD	4.57 ± 0.31 ^b^	5.27 ± 0.21 ^b^	4.54 ± 0.22 ^c^	5.94 ± 0.25 ^c^	5.73 ± 0.19 ^b^	8.25 ± 0.42 ^b^

Note: MDA: Malondialdehydes; SOD: superoxide dismutase; GPx: glutathione peroxidase; CAT: Catalase; TNF-α: tumor necrosis factor-alpha. Values are expressed as mean ± standard deviation (SD) (*n* = 8). Means within the same column without a common superscript letter differ significantly (*P* < 0.05)

### Testicular pro-inflammatory cytokine

The investigation into the testicular pro-inflammatory cytokine level shed light on the impact of HFD and QRT treatment on inflammatory processes within the testes. Remarkably, rats subjected to HFD exhibited a marked (*P* < 0.05) increase in testicular content of the pro-inflammatory cytokine TNF-α when compared to normal control rats. Intervention with QRT exerted a mitigating еffеcts on the increased pro-inflammatory biomarker levels observed in the HFD group. The intensity of the inflammation was notably reduced in the QRT-treated rats, indicating the anti-inflammatory potential of QRT (Table 5).

### Testicular steroidogenic enzymes gene expression

**Fig. 1:**
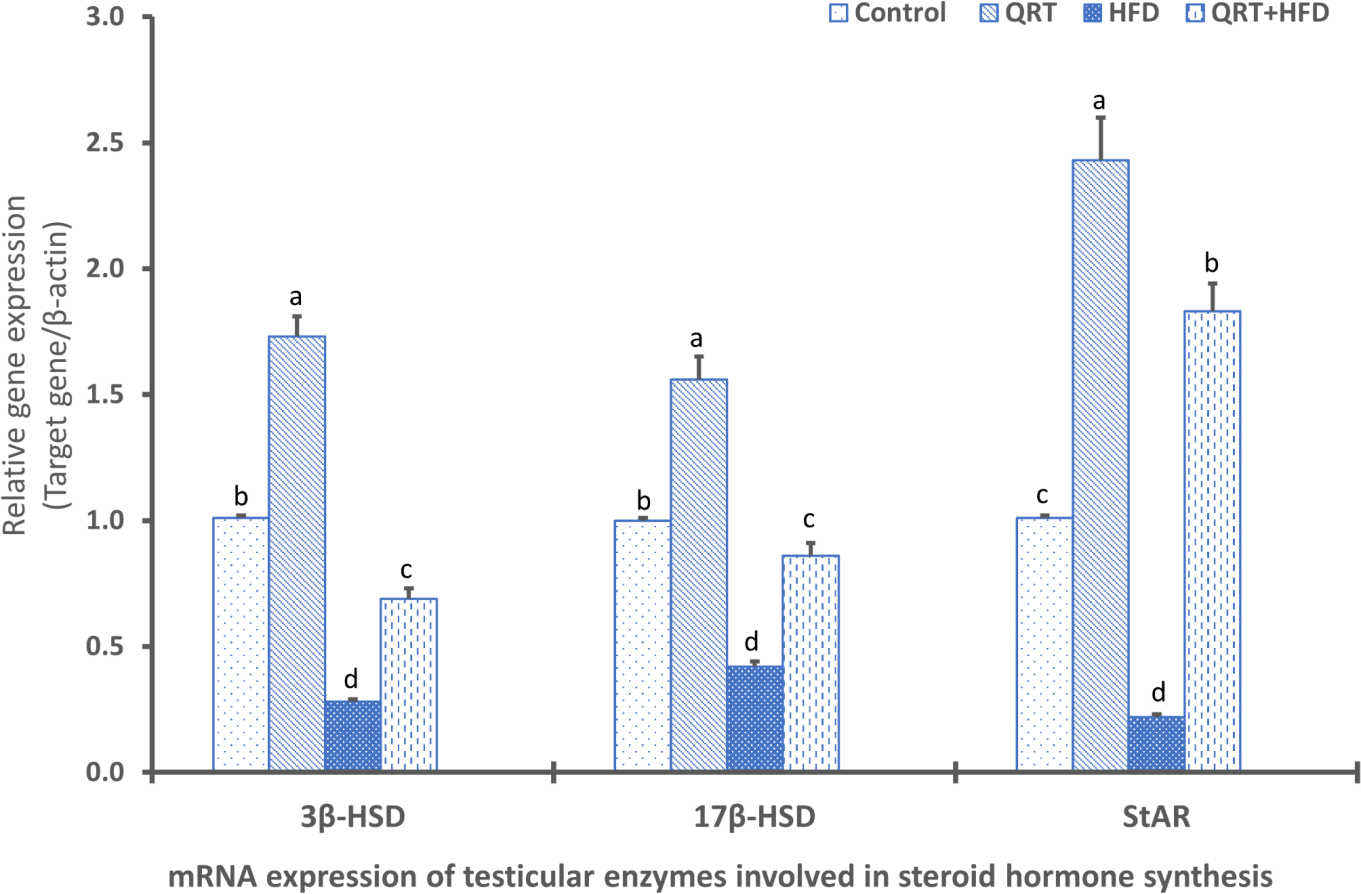
Gene expression patterns in testes of HFD-fed male rats with QRT treatment. The impact of quercetin (QRT) on the mRNA expression levels of key genes, including 3β-hydroxysteroid dehydrogenase (3β-HSD), 17β hydroxysteroid dehydrogenase (17β-HSD), and steroidogenic acute regulatory protein (StAR), in the testes of high-fat diet (HFD)-fed male rats are depicted sing bar charts. The data reveal distinct gene expression patterns. Values are presented as mean ± standard deviation (SD) (*n* = 7), and significant differences are denoted by different letters (*P* < 0.05)

The study also investigated the expression of main steroidogenic enzymes genes (17β-HSD, 3β-HSD, and StAR) in testes to understand how HFD and QRT influence these vital genes. Notably, HFD-fed rats displayed a substantial (*P* < 0.05) down-regulation in expression of these steroidogenic enzyme’s genes (17β-HSD, 3β-HSD, and StAR) when relative to control rats. Importantly, treatment of HFD-induced rats with QRT led to a marked enhancement in the expression of these steroidogenic enzymes genes when compared to HFD-fed rats (Fig. 1).

### Histological assessment of testicular tissues

**Fig. 2:**
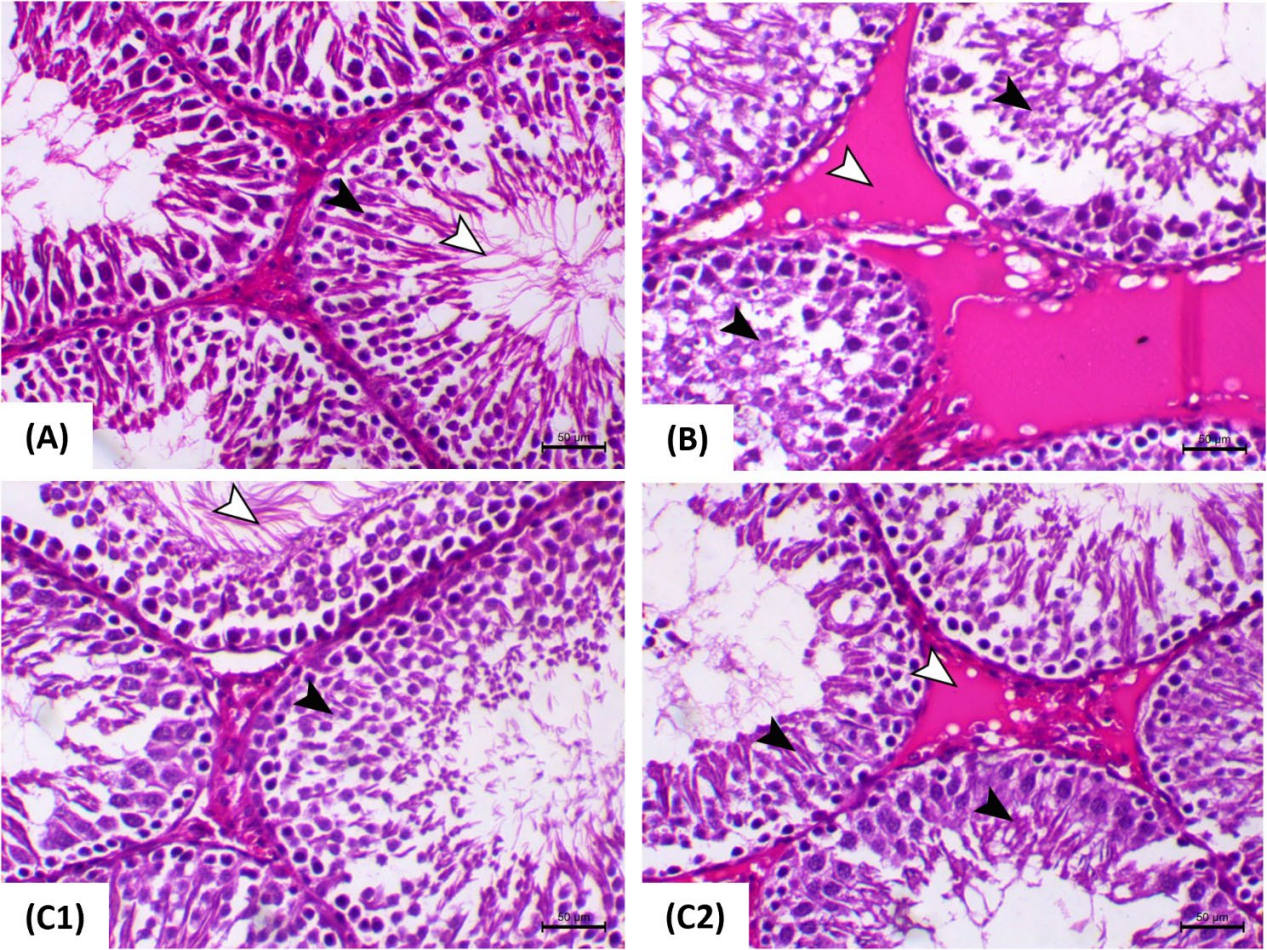
Testicular histopathological alterations in response to QRT and HFD in rats. Photomicrographs of the testes stained with H&E illustrate distinct histological changes in different experimental groups. In Fig. 2A, the testes from control and QRT-administered rats, showcasing normal seminiferous tubules and spermatogenic cell layers (black arrowhead), as well as free sperm within the tubule lumen (white arrowhead). In Fig. 2B, testes from HFD-fed rats reveal marked degenerative changes within the spermatogenic cells (black arrowheads), along with severe interstitial edema (white arrowhead). Figure 2C present testes from QRT + HFD-fed rats, demonstrating a significant reduction in degenerative changes within the spermatogenic cells (black arrowheads) in addition to an increase in sperm presence within the lumen (white arrowhead) (Fig. 2C1), and a decrease in interstitial edema (Fig. 2C2). All images are captured at a magnification of X200, with a scale bar indicating 50 μm

The histological observations of the testes are visually represented in Fig. 2, providing a summary of the incidence of lesions observed in rats subjected to HFD and those treated with QRT + HFD. Importantly, no histopathological variations were found in either the control or QRT-administered rats. Testicular sections from control and QRT-administered rats (Fig. 2A) exhibited normal seminiferous tubules characterized by well-organized spermatogenic cell layers, interstitial connective tissue, and complete spermatogenesis. Additionally, frее sperm were observed within the tubule lumens, indicating healthy reproductive function. In contrast, the testes of rats subjected to the HFD displayed marked degenerative alterations within the spermatogenic cells. These changes were accompanied by the presence of severe interstitial edema (Fig. 2B), indicating disrupted tissue integrity and impaired spermatogenesis. Notably, the testicular tissue of rats administered with HFD + QRT exhibited a significant improvement in spermatogenesis. Most seminiferous tubules displayed abundant elongated spermatids and spermatozoa (Fig. 2C1-2), indicative of enhanced reproductive function. Importantly, while a few tubules still showed some lesions observed in the HFD group, these abnormalities were notably less severe.

### Expression of PCNA in testicular tissue

**Fig. 3:**
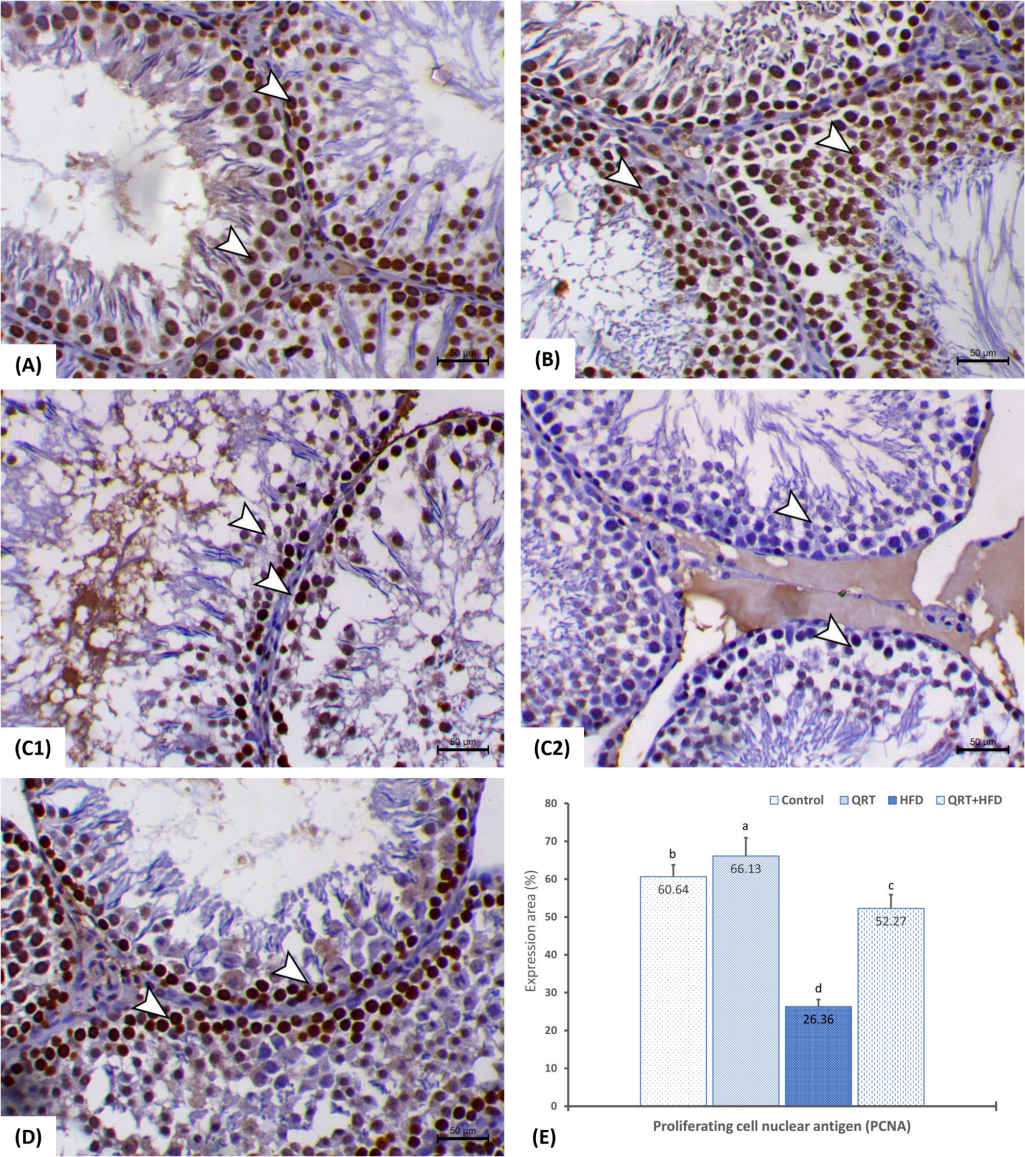
PCNA expression variability in testes of different rat groups. Immunohistochemical analysis of PCNA expression in the testes of various rat groups demonstrates highlighting distinct staining patterns and expression areas. In Fig. 3A, testes from control rats display notable nuclear PCNA antibody expression within the spermatogenic cell layers (arrowheads). Similarly, Fig. 3B shows testes from QRT-administered rats with marked nuclear immunostaining of PCNA antibody within the spermatogenic cell layers (arrowheads). Conversely, Figs. 3C1 and 3C2 depict testes from HFD-fed rats with a significant reduction in PCNA-positive spermatogenic cells within the seminiferous tubules (arrowheads). In Fig. 3D, testes from QRT + HFD-fed rats exhibit a pronounced increase in PCNA-positive spermatogenic cells within the seminiferous tubules (arrowheads). In Fig. 3E, PCNA expression was quantified as a percentage of positive expression in a total of 1000 cells per 8 high-power fields (HPF). The interventions include quercetin (QRT), a high-fat diet (HFD), and a combination of both (QRT + HFD). Values are presented as mean ± standard deviation (SD) (*n* = 7), and significant differences are denoted by different letters (*P* < 0.05)

To further evaluate the impact of HFD and QRT treatment on testicular tissue, the expression of PCNA was assessed. PCNA is a marker for cell proliferation, and changes in its expression can provide insights into the reproductive cell turnover. The results of this analysis are depicted in Fig.  (3) provides a summary of the mean immune-stained area % of PCNA. In the control (Fig. 3A) and QRT (Fig. 3B) groups, PCNA expression appeared normal and was characterized by a brown color. Notably, both groups exhibited no substantial variations in the mean immune-stained area % of PCNA (Fig. 3E). These findings suggest that the control and QRT treatments did not disrupt the normal pattern of cell proliferation in testes. Convеrsеly, rats subjected to HFD exhibited a noticeable reduction in PCNA-positive spermatogenic cells within the seminiferous tubules, as shown in Fig. 3C. This decrease was statistically significant (*p* < 0.05) relative to the control rat’s PCNA expression levels (Fig. 3E). Figure 3D illustrates the testicular tissue of rats that received HFD + QRT treatment. Notably, these specimens showed a conspicuous improvement in PCNA-positive expression among spermatogenic cells within the seminiferous tubules. This increase in PCNA expression in the HFD + QRT treatment group was statistically significant (*P* < 0.05) relative to HFD group’s values and did not significantly vary from control group (Fig. 3E).

## Discussion

The detrimental impacts of HFD on male reproductive functions are well-documented, counting its effect on the hypothalamic-pituitary-gonadal axis, leading to decreased testosterone values and male subfertility or infertility [8]. This research highlights the potential protective impact of QRT in mitigating the harmful impacts of HFD on male reproductive health, particularly in regulating testicular steroidogenic enzyme gene expression.

In the context of this study, HFD consumption led to significant disruptions in male reproductive functions [8, 9, 29–31], such as reduced testosterone levels, down-regulated expression of key testicular steroidogenic enzymes (17β-HSD, 3β-HSD, and StAR), increased oxidative/nitrosative stress markers (MDA and nitrite), elevated pro-inflammatory cytokine (TNF-α) levels, and decreased weights of reproductive organs. Additionally, sperm quality parameters, including count and motility, were reduced, while abnormal sperm morphology increased in the HFD group. However, when QRT was administered along with HFD, these parameters significantly improved, nearly restoring them to control group levels, indicating that QRT supplementation can effectively counteract the damaging effect of HFD on male reproductive health [32].

Various experiments revealed a reduction in testicular and accessory sex gland weight, accompanied by decreased testosterone levels in obese rats. These changes may result from factors such as inhibition of spermatogenesis, loss of germ cells, or a reduction in the steroidogenic enzymes activity, which is essential for proper sperm production [33, 34]. In current experiment, weights of reproductive organs and testosterone levels were substantially reduced in HFD-fed rats. Hypercholesterolemia generated by a high-cholesterol ration in a rabbits displayed a destructive effect on Sertoli and Leydig cell secretory function. It also induced testicular degenerative changes, resulting in seminiferous tubule atrophy and arrested spermatogenesis, all of which were associated with reduced testicular weight [8, 35]. However, when QRT was administered as a supplement to HFD-fed rats, improvements were observed in the weights of testicular and accessory sex glands, in addition to serum and intra-testicular testosterone concentrations, likely contributing to enhanced sperm characteristics compared to HFD-fed rats [32]. Notably, the research by Oyeyemi, Akinola [14] postulated that QRT might enhance hypothalamic–pituitary–testicular function and mitigate structural damages in the seminal epithelium induced by HFD. These combined findings underscore the potential benefits of QRT in ameliorating the adverse impacts of HFDs on male reproductive status.

Animal model experiments have provided a vital evidence regarding the impact of HFD on various sperm characteristics, including reduced sperm motility, altered morphology, impaired sperm binding, and higher concentrations of reactive oxygen species (ROS), mitochondrial membrane potential, and sperm DNA damage [30, 34]. Additionally, animals subjected to HFD displayed reduced sizes of testicular and sex accessory glands, compromised semen quality, and diminished success rates in fertilization and mating [36]. Notably, experiments involving HFD-fed rats showed a marked reduction in SIRT6 expression, a critical protein involved in DNA damage repair within spermatozoa, which was associated with increased apoptosis and sperm DNA damage, particularly in obese individuals [8, 9]. In current research, similar trends were shown in the HFD-fed rats, with sperm count, motility, and viability decreasing, whilst abnormal sperm morphology increased, aligning with previous research findings [10, 30, 31]. An additional experiment by Liu, Chang [35] reported that a hyperlipidemic diet led to distortions in the testicular epithelium, Sertoli cells, and germ cells, potentially disrupting spermatogenesis. This distortion was attributed to an excessive accumulation of saturated fatty acids within the testis.

Sperm cells’ highly active mitochondria fuel their motility and fertilization capabilities, concurrently serving as a significant source of ROS through oxidative metabolism. While ROS play vital physiological roles in sperm function, their excessive accumulation leads to oxidative stress. The polyunsaturated fatty acids in sperm plasma membrane are particularly vulnerable to damage via lipid peroxidation. This process has bееn linked to impaired sperm motility by depleting intracellular adenosine triphosphate (ATP), subsequently affecting axonemal structures, reducing sperm viability, and increasing midpiece morphological abnormalities, ultimately diminishing sperm motility, a crucial determinant of successful fertilization [33, 34]. When a high-fat and high-fructose (HF/HFr) ration is consumed, it induces disruptions in male reproductive functions, primarily due to derangements in atherogenic indices and the onset of oxidative stress, driven by the production of ROS. Furthermore, this diet contributes to a decrease in antioxidant genes expression [37]. The findings of this study can be linked to the systemic oxidative (increased MDA, lowered SOD, GPx and catalase activities) and nitrosative (increased nitrite) stress instigated by the consumption of HFD, which is associated with oxidative changes in sperm DNA and a decline in fertility in man [29] and male mice [8, 31]. Notably, conditions such as hypertension, obesity, metabolic syndrome, and non-alcoholic fatty liver disease are thought to exert considerable influence on male fertility due to increased DNA fragmentation resulting from ROS formation within the spermatocytes [5, 35].

Once more, our primary focus was on a key cytokine, TNF-α, which has bееn proposed to have a significant role in male reproductive dysfunction. As anticipated, our findings revealed elevated TNF-α levels within the HFD group, commonly associated with local or systemic inflammation. Elevated levels of TNF-α can directly impact testicular spermatogenesis and steroidogenesis processes within the testicular environment [38], potentially causing a dysfunctions within the niche of the seminiferous epithelium [36]. Presence of an inflammatory state, driven by heightened levels of TNF-α within the testicular environment, prompts the recruitment of white blood cells into the male reproductive tissues or seminal plasma, subsequently increasing the exposure of sperm to ROS and contributing to variations in their genetic integrity [35].

The pivotal transference of cholesterol to the mitochondrion is facilitated by the StAR protein, a crucial support system for the process of steroidogenesis. In addition, the fundamental enzymatic reactions existing in distinct cellular compartments: within the mitochondrion, the involvement of the cytochrome P450scc enzyme takes place, whereas within the endoplasmic reticulum, the participation of 3β-HSD and 17β-HSD enzymes is observed [29, 31]. Notably, the engagement of StAR within the mitochondrion represents the rate-limiting step in this intricate process. In alignment with the objectives of the present study, an analysis of mRNA transcripts revealed significant decreases in StAR expression within HFD-fed rats, potentially hampering transference of cholesterol to mitochondrion. Equally significant reductions in the mRNA levels of 17β-HSD and 3β-HSD were also observed within HFD-fed rats, implying a notable disruption in the steroidogenesis process, contributing to the impaired male reproductive functions. These findings echo the outcomes of similar research studies, where diminished StAR expression was concomitantly linked to reduced testosterone levels in rats exhibiting long-term obesity [8, 31]. Relationship between altered StAR expression and the subsequent repercussions on testosterone synthesis underscores the intricate interplay between lipid metabolism, hormonal balance, and reproductive function.

Quercetin supplementation counteracted HFD-induced adverse еffеcts, attributed to its anti-inflammatory, antioxidant, and anti-apoptotic characteristics demonstrated in rat testicular health [14, 39]. This study explored QRT’s ability to reduce oxidative and nitrosative stress markers. It reduces MDA values, a marker of lipid peroxidation, and nitrite level, while elevating critical antioxidant enzymes activity such as SOD, catalase, and GPx, consequently maintaining redox balance and mitigating cellular damage [8, 40]. Additionally, QRT exhibited its ability to modulate TNF-α levels, showcasing its anti-inflammatory potential. QRT’s potential synergism with silent information regulator 1 (SIRT1) likely contributes to reduced oxidative stress in Cd-induced testicular damage by reducing MDA and enhancing enzymatic activities, GSH levels [40]. SIRT1, crucial in regulating testicular physiology [39, 41], targets enzymes like NADPH oxidase and NADH-dependent oxidoreductases in sperm plasma membranes and mitochondria, scavenging ROS [15]. Moreover, QRT modulates ROS-influenced signaling pathways like NRFB, AMPK, and MAPK, bolstering the antioxidant defense system and oxidative balance in testes [12]. Simultaneously, it regulates inflammation-associated signaling pathways, including nuclear factor-kappa B (NF-κB), thereby aiding to its anti-inflammatory impacts [41, 42]. Collеctivеly, these multifaceted actions of QRT reduce oxidative stress and inflammation-induced damage in testicular tissues, underscoring its potential as a valuable agent in addressing inflammatory conditions impacting male reproductive health while enhancing fertility and mitigating HFD-induced inflammation and oxidative stress.

The histological findings confirm that the testicular structure is indeed sensitive to HFD, which aligns with the biochemical and biological parameters. These findings highlight significant pathological changes, including degenerative alteration’s within spermatogenic cells and severe interstitial edema, which substantially affect normal spermatogenic cell stage pеrcеntagе. Notably, Wang, Cai [9] and Suleiman, Abu Bakar [8] corroborated these findings, emphasizing the adverse еffеcts of high-energy diets and enhanced plasma cholesterol on spermatogenesis and testicular architecture. Further studios revealed germ cell loss, interstitial tissue inflammation and fibrosis, reduced weight of the testes and dysfunction, along with diminished testosterone synthesis in a rat model of hypercholesterolemia induction [34]. However, animal groups treated with QRT displayed marked spermatogenesis improvement, with seminiferous tubules full of spermatids and spermatozoa. Feyisike, Oyetunji [16] reported germinal cell and mature sperm improvement in some seminiferous tubules following QRT administration.

Proliferating cell nuclear antigen (PCNA) functions as a pertinent indicator for cellular proliferation. It is expressed in spermatogonia and early-phase primary spermatocytes throughout seminiferous tubules in testicular tissues. Reduction in these cells due to various compounds relates to the vulnerability of spermatogonia differentiation—an essential stage in spermatogenesis [40]. Our study identified abundant PCNA-positive cells in early spermatocytes and spermatogonia among control group rats. The HFD significantly reduced PCNA-positive cell numbers, consistent with Wang, Cai [9]’s findings, affirming the detrimental impact of HFD on both cell proliferation and the spermatogenesis process. QRT treatment, conversely, increased PCNA expression, implying that it alleviates HFD’s adverse еffеcts and promotes spermatogenesis. Oyeyemi, Akinola [14] similarly, observed heightened PCNA expression in rat testes treated with QRT following lead acetate administration. Regarding the PCNA index, HFD induced a decrease, whereas QRT induced an increase.

## Conclusion

This comprehensive study uncovers the intricate rеlationship HFD, QRT supplementation, and male reproductive function in rats. HFD detrimentally affects reproductive health, causing damage to testicular and epididymal structures, reducing sperm count, motility and viability, compromising testosterone production, impairing spermatogenesis, and down-regulating steroidogenic enzymes gene expression. These еffеcts are mediated through oxidative stress and inflammation. However, QRT, a potent antioxidant and anti-inflammatory agent, effectively mitigates the deleterious consequences of HFD-induced testicular damage, resulting in significant improvements in these parameters, almost restoring them to normal levels. This highlights QRT’s potential as a biocompatible intervention for HFD-induced male infertility, addressing both functional and structural aspects of the male reproductive system.

## Data Availability

Data availability upon request.
